# Expected immune recognition of COVID-19 virus by memory from earlier infections with common coronaviruses in a large part of the world population

**DOI:** 10.12688/f1000research.23458.2

**Published:** 2020-07-17

**Authors:** Johannes M. Dijkstra, Keiichiro Hashimoto

**Affiliations:** 1 Institute for Comprehensive Medical Science, Fujita Health University, Toyoake, Aichi, 470-1192, Japan

**Keywords:** Coronavirus, COVID-19, SARS-CoV-2, OC43, HKU1, 229E, NL63, MHC class I, Immunology, Vaccination

## Abstract

SARS-CoV-2 is the coronavirus agent of the COVID-19 pandemic causing high mortalities. In contrast, the widely spread human coronaviruses OC43, HKU1, 229E, and NL63 tend to cause only mild symptoms. The present study shows, by *in silico* analysis, that these common human viruses are expected to induce immune memory against SARS-CoV-2 by sharing protein fragments (antigen epitopes) for presentation to the immune system by MHC class I. A list of such epitopes is provided. The number of these epitopes and the prevalence of the common coronaviruses suggest that a large part of the world population has some degree of specific immunity against SARS-CoV-2 already, even without having been infected by that virus. For inducing protection, booster vaccinations enhancing existing immunity are less demanding than primary vaccinations against new antigens. Therefore, for the discussion on vaccination strategies against COVID-19, the available immune memory against related viruses should be part of the consideration.

## Introduction

### SARS-CoV-2 and other human coronaviruses

From the end of 2019, the world experienced the coronavirus disease 2019 (COVID-19) pandemic caused by the emerging severe acute respiratory syndrome coronavirus 2 (SARS-CoV-2; aka 2019 novel coronavirus or 2019-nCoV). SARS-CoV-2 shares ~80% nucleotide identity with SARS-CoV-1 (aka SARS-CoV), the causative agent of the SARS epidemy from 2002, and is even more similar to some coronaviruses in bats ( Andersen *et al.*, 2020; Ceraolo & Giorgi, 2020; Wu *et al.*, 2020; Zhou *et al.*, 2020). Coronaviruses are membrane-enveloped positive-strand RNA viruses with, for an RNA virus, a large genome of ~30 kb. That genome encodes several structural components of the virion including the nucleocapsid protein N and the membrane proteins S (spike), M, and E, plus also a number of nonstructural proteins involved in RNA replication and other—partly unknown—functions ( Weiss & Navas-Martin, 2005). The coronaviruses infecting humans belong to the serological/phylogenetic clades group I (alphacoronaviruses) and group II (betacoronaviruses); group I includes HCoV-229E (human coronavirus 229E) and HCoV-NL63, while group II includes SARS-CoV-1, SARS-CoV-2, Middle East respiratory syndrome coronavirus (MERS-CoV), HCoV-OC43, and HCoV-HKU1. The viruses SARS-CoV-1 and MERS-CoV, on average, cause the most severe symptoms, and their outbreaks were successfully monitored and halted. At the other end of the spectrum, the viruses HCoV-229E, HCoV-NL-63, HCoV-OC43, and HCoV-HKU1 tend to cause only mild symptoms and are very common.

### Prevalence and associated disease of the common human coronaviruses 229E, NL63, OC43, and HKU1

The Centers for Disease Control and Prevention (CDC; https://www.cdc.gov/coronavirus/general-information.html) states: “Common human coronaviruses, including types 229E, NL63, OC43, and HKU1, usually cause mild to moderate upper-respiratory tract illnesses, like the common cold. Most people get infected with one or more of these viruses at some point in their lives.” The same agency lists the common symptoms caused by these viruses as runny nose, sore throat, headache, fever, cough, and general feeling of being unwell, but also explains that they occasionally cause lower-respiratory tract illnesses, such as pneumonia or bronchitis. The viruses 229E and OC43 have been known since the 1960s (reviewed in Kahn & McIntosh, 2005), but NL63 ( van der Hoek *et al.*, 2004) and HKU1 ( Woo *et al.*, 2005) were only (conclusively) identified following the rise in interest in coronaviruses in the wake of the SARS epidemy. These common coronaviruses are believed to be the second most common cause of the common cold ( Mäkelä *et al.*, 1998). In the U.S.A., a 3-year RT-PCR surveillance of respiratory samples of patients revealed that the four viruses 229E, NL63, OC43, and HKU1 were present at levels varying by season and region, with all individual viruses peaking at >3% prevalence in each investigated region (Midwest, Northeast, South, West); co-infection with other coronaviruses was found in only ~2% of infected cases, but co-infection with another respiratory virus was found in a substantial ~30% of infected cases ( Killerby *et al.*, 2018). This pattern was reminiscent of findings in the United Kingdom ( Gaunt *et al.*, 2010) and Japan ( Matoba *et al.*, 2015). Serological investigations in countries as diverse as the U.S.A. ( Bradburne & Somerset, 1972; Dijkman *et al.*, 2012), China ( Zhou *et al.*, 2013), and Qatar ( Al Kahlout *et al.*, 2019), found that most healthy blood donors had antibodies against coronaviruses, supporting that these viruses are widespread indeed.

Since immune memory protection can be induced by related pathogens, as exemplified by the eradication of human smallpox virus (Variola) by immunization with a related “cowpox” virus (Vaccinia) ( Plotkin & Plotkin, 2018), it is interesting to consider whether common human coronavirus infections may have induced some level of protection against SARS-CoV-2.

### The possibility of matching linear epitopes between SARS-CoV-2 and the common human coronaviruses that may stimulate the immune system through MHC class I presentation

The major arms of immune memory concern antibody secretion by B cells, killing of infected cells by CD8 ^+^ T cells, and helper/regulatory immune activities (e.g. cytokine secretion) by CD4 ^+^ T cells. For a murine coronavirus infection in mouse, both antibody responses and cell-mediated cytotoxicity were needed to efficiently control the virus (reviewed by Weiss & Navas-Martin, 2005). In SARS-CoV-1-infected patients, B cell as well as T cell responses were observed ( Li *et al.*, 2008), and, in animal models of SARS, B cell responses ( Bisht *et al.*, 2005) as well as CD4 ^+^ and CD8 ^+^ T cell responses ( Channappanavar *et al.*, 2014; Liu *et al.*, 2017; Zhao *et al.*, 2010; Zhao *et al.*, 2016) were shown to have protective value. Notably, in most individuals who had recovered from SARS, SARS-CoV-1-specific memory CD8 ^+^ T cells persisted for up to 6 years after SARS-CoV-1 infection whereas memory B cells and antivirus antibodies generally became undetectable ( Tang *et al.*, 2011).

Based on theoretical considerations alone, it is difficult to predict effective B cell memory across different virus species ( Qiu *et al.*, 2020), which makes it a poor topic for our present study which is based on sequence comparisons. We just note that a recent study concluded that sera from people that likely had been infected with the common human coronaviruses 229E, NL63, OC43, and/or HKU1, possessed no or negligible cross-reactivity with SARS-CoV-2 virus S protein ( Amanat *et al.*, 2020) and thus probably possess no neutralizing antibodies.

There may be some recognition of SARS-CoV-2 epitopes by CD4 ^+^ T cell memory derived from previous infections with common human coronaviruses. However, as discussed in the Results and Discussion section, the very limited lengths of identical sequence stretches between the viruses make theoretical predictions of such epitopes difficult, and therefore the current study only concentrates on potential CD8 ^+^ T cell memory.

For inducing CD8 ^+^ T cell memory, the core requirement is merely that an identical short peptide is presented by major histocompatibility complex (MHC) class I (MHC-I) molecules. MHC-I molecules present peptide fragments from intracellular proteins, thus also from viral proteins, at the cell surface for screening by CD8 ^+^ cytotoxic T cells ( Neefjes *et al.*, 2011). CD8 ^+^ T cells recognize the combination of MHC-I molecule with peptide by T cell receptors (TCR) that are unique per T cell clone, and if stimulated these clones can proliferate, kill the presenting (virus-infected) cell, and produce memory cells. MHC-I molecules are polymorphic in that they are represented by many diverse allelic forms that differ between human populations and individuals ( Robinson *et al.*, 2020), and mostly bind peptides of 9 amino acids (aa) length in their binding groove which is closed at either end ( Bjorkman *et al.*, 1987; Rammensee *et al.*, 1995; Schellens *et al.*, 2015).

In the present study, we analyzed whether there are linear 9 aa epitopes that are identical between proteins encoded by SARS-CoV-2 and one or more of the common human coronaviruses. We found many of such epitopes indeed, and, by using prediction software, found that some are expected to bind well to certain MHC-I alleles. We therefore expect that common human coronaviruses can induce some level of CD8 ^+^ T cell-mediated immune memory recognizing SARS-CoV-2, and consider the possibility of enhancing that immune memory by vaccination.

## Methods

Proteins encoded by a reported genomic sequence for SARS-CoV-2 (GenBank MN908947; Wu *et al.*, 2020) were compared with those for HCoV-OC43 (NC_005147; Vijgen *et al.*, 2005), HCoV-HKU1 (NC_006577; Woo *et al.*, 2005), HCoV-229E (NC_002645; Thiel *et al.*, 2001), and HCoV-NL63 (NC_005831; van der Hoek *et al.*, 2004) by performing BLAST homology searches at the NCBI database ( https://blast.ncbi.nlm.nih.gov/Blast.cgi) and by making multiple sequence alignments using CLUSTALW software ( https://www.genome.jp/tools-bin/clustalw); continuous stretches of 9 aa acids identical between SARS-CoV-2 and one of the other viruses were identified manually. All these shared 9 aa epitopes were screened by ANN 4.0 software at IEDB Analysis Resource ( http://tools.immuneepitope.org/mhci/) for prediction of their affinity to a set of representative human MHC-I alleles.

## Results and discussion

Table 1 lists the 9 aa epitopes that are identical between proteins encoded by SARS-CoV-2 and one or more of the common human coronaviruses. Many identical >9 aa stretches were found with ORF1ab encoded polyprotein, one such identical stretch (of 12 aa) was found with the N protein of the other two type II coronaviruses HCoV-OC43 and HCoV-HKU1, and no such stretches were found when comparing with any of the other gene products; ORF1ab-derived mature proteins with such stretches, expected from cleavage of the polyprotein precursor ( Wu *et al.*, 2020), were the transmembrane protein nonstructural protein 4 (NSP4), 3C-like cysteine protease NSP5, RNA binding protein NSP9, RNA dependent RNA polymerase NSP12, helicase NSP13, 3’-to-5’ exonuclease NSP14, nidoviral endoribonuclease specific for U NSP15, and S-adenosylmethionine-dependent ribose 2’-O-methyltransferase NSP16 ( Table 1). Sequence alignment figures of the ORF1ab and N proteins are shown in *Extended data* ( Dijkstra, 2020a) with highlighting of the interesting epitopes. It is of note that the S protein, which is the prime candidate for inducing neutralizing antibodies ( Cohen, 2020), is poorly suitable for inducing an MHC-I-restricted immune memory across the investigated viral species as between S protein of SARS-CoV-2 and S proteins of the common human coronaviruses there are no 9 aa matches, and, among the virus isolates compared in this study, only a single 8 aa match (DRLITGRL with HCoV-NL63 and -229E) (not shown).

**Table 1.** Stretches of 9 consecutive amino acids that are identical between SARS-CoV-2 and at least one of the common human coronaviruses. Compared sequences were derived from the following GenBank accessions: for ORF1ab SARS-CoV-2, QHD43415; OC43, NP_937947; HKU1, YP_173236; 229E, NP_073549; NL63, YP_003766; and for N SARS-CoV-2, QHD43423; OC43, NP_937954; HKU1, YP_173242; 229E, NP_073556; NL63, YP_003771 Source positions indicate the N-terminal position of the depicted 9 aa sequence in the SARS-CoV-2 ORF1ab protein or N protein (see Supplementary file 1). The ORF1ab protein is only a precursor polyprotein, and the column Mature protein indicates the probable mature protein that possesses the epitope: 3CLpro, 3C-like cysteine protease; RdRp, RNA dependent RNA polymerase; Hel, helicase; ExoN, 3’-to-5’ exonuclease; NendoU, nidoviral endoribonuclease specific for U; O-MT, S-adenosylmethionine-dependent ribose 2’-O-methyltransferase. Yellow blocks indicate the presence of identical sequences in the respective common human coronavirus, and gray blocks indicate the absence of such matches. Orange blocks highlight those peptides with predicted IC50 values of <500 nM for one of the twelf investigated MHC-I alleles. No predicted IC50 values of <500 nM were found for HLA-C*0401

			common human coronaviruses				IC50 prediction by ANN 4.0 program of IEDB software (only IC50 valuesof <500 nM are shown)													
	Mature		Group II		Group I		HLA-A					HLA-B							HLA-C	
SARS-CoV-2 source	protein	Sequence (9aa)	OC43	HKU1	229E	NL63	*0101	*0201	*0301	*2402	*2601	*0702	*0801	*1501	*2705	*3901	*4001	*5801	*0303	*0401
ORF1ab 3004	NSP4	WVLNNDYYR																		
ORF1ab 3005	NSP4	VLNNDYYRS																		
ORF1ab 3006	NSP4	LNNDYYRSL																		
ORF1ab 3007	NSP4	NNDYYRSLP																		
ORF1ab 3008	NSP4	NDYYRSLPG																		
ORF1ab 3289	NSP5 3CLpro	TLNGLWLDD																		
ORF1ab 3299	NSP5 3CLpro	VYCPRHVIC																		
ORF1ab 4208	NSP9	ELEPPCRFV																		
ORF1ab 4551	NSP12 RdRp	KKDWYDFVE																		
ORF1ab 4552	NSP12 RdRp	KDWYDFVEN																		
ORF1ab 4553	NSP12 RdRp	DWYDFVENP																		
ORF1ab 4554	NSP12 RdRp	WYDFVENPD																		
ORF1ab 4555	NSP12 RdRp	YDFVENPDI																		
ORF1ab 4594	NSP12 RdRp	VGVLTLDNQ																		
ORF1ab 4595	NSP12 RdRp	GVLTLDNQD																		
ORF1ab 4596	NSP12 RdRp	VLTLDNQDL																		
ORF1ab 4597	NSP12 RdRp	LTLDNQDLN																		
ORF1ab 4598	NSP12 RdRp	TLDNQDLNG																		
ORF1ab 4599	NSP12 RdRp	LDNQDLNGN																		
ORF1ab 4608	NSP12 RdRp	WYDFGDFIQ																		
ORF1ab 4609	NSP12 RdRp	YDFGDFIQT																		
ORF1ab 4661	NSP12 RdRp	DLLKYDFTE																		
ORF1ab 4725	NSP12 RdRp	IFVDGVPFV						158 nM												
ORF1ab 4726	NSP12 RdRp	FVDGVPFVV						10 nM								143 nM				
ORF1ab 4727	NSP12 RdRp	VDGVPFVVS																		
ORF1ab 4799	NSP12 RdRp	FQTVKPGNF																		
ORF1ab 4800	NSP12 RdRp	QTVKPGNFN																		
ORF1ab 4931	NSP12 RdRp	TQMNLKYAI						278 nM								21 nM				
ORF1ab 4932	NSP12 RdRp	QMNLKYAIS																		
ORF1ab 4933	NSP12 RdRp	MNLKYAISA																		
ORF1ab 4934	NSP12 RdRp	NLKYAISAK							198 nM											
ORF1ab 4935	NSP12 RdRp	LKYAISAKN																		
ORF1ab 4936	NSP12 RdRp	KYAISAKNR																		
ORF1ab 4937	NSP12 RdRp	YAISAKNRA																		
ORF1ab 4938	NSP12 RdRp	AISAKNRAR																		
ORF1ab 4939	NSP12 RdRp	ISAKNRART																		
ORF1ab 4940	NSP12 RdRp	SAKNRARTV											58 nM							
ORF1ab 4941	NSP12 RdRp	AKNRARTVA																		
ORF1ab 4942	NSP12 RdRp	KNRARTVAG																		
ORF1ab 4943	NSP12 RdRp	NRARTVAGV													468 nM					
ORF1ab 4944	NSP12 RdRp	RARTVAGVS																		
ORF1ab 4945	NSP12 RdRp	ARTVAGVSI													219 nM					
ORF1ab 5006	NSP12 RdRp	LMGWDYPKC																		
ORF1ab 5007	NSP12 RdRp	MGWDYPKCD																		
ORF1ab 5008	NSP12 RdRp	GWDYPKCDR																		
ORF1ab 5009	NSP12 RdRp	WDYPKCDRA																		
ORF1ab 5010	NSP12 RdRp	DYPKCDRAM																		
ORF1ab 5011	NSP12 RdRp	YPKCDRAMP																		
ORF1ab 5012	NSP12 RdRp	PKCDRAMPN																		
ORF1ab 5043	NSP12 RdRp	RFYRLANEC																		
ORF1ab 5044	NSP12 RdRp	FYRLANECA																		
ORF1ab 5045	NSP12 RdRp	YRLANECAQ																		
ORF1ab 5046	NSP12 RdRp	RLANECAQV						53 nM												
ORF1ab 5047	NSP12 RdRp	LANECAQVL																	26 nM	
ORF1ab 5048	NSP12 RdRp	ANECAQVLS																		
ORF1ab 5049	NSP12 RdRp	NECAQVLSE																		
ORF1ab 5066	NSP12 RdRp	YVKPGGTSS																		
ORF1ab 5067	NSP12 RdRp	VKPGGTSSG																		
ORF1ab 5068	NSP12 RdRp	KPGGTSSGD																		
ORF1ab 5069	NSP12 RdRp	PGGTSSGDA																		
ORF1ab 5070	NSP12 RdRp	GGTSSGDAT																		
ORF1ab 5071	NSP12 RdRp	GTSSGDATT																		
ORF1ab 5072	NSP12 RdRp	TSSGDATTA																		
ORF1ab 5074	NSP12 RdRp	SGDATTAYA																		
ORF1ab 5075	NSP12 RdRp	GDATTAYAN																		
ORF1ab 5076	NSP12 RdRp	DATTAYANS																		
ORF1ab 5077	NSP12 RdRp	ATTAYANSV																		
ORF1ab 5078	NSP12 RdRp	TTAYANSVF									245 nM			29 nM						
ORF1ab 5079	NSP12 RdRp	TAYANSVFN																	466 nM	
ORF1ab 5080	NSP12 RdRp	AYANSVFNI								56 nM										
ORF1ab 5082	NSP12 RdRp	ANSVFNICQ																		
ORF1ab 5083	NSP12 RdRp	NSVFNICQA																		
ORF1ab 5084	NSP12 RdRp	SVFNICQAV						85 nM												
ORF1ab 5085	NSP12 RdRp	VFNICQAVT																		
ORF1ab 5086	NSP12 RdRp	FNICQAVTA																		
ORF1ab 5087	NSP12 RdRp	NICQAVTAN																		
ORF1ab 5088	NSP12 RdRp	ICQAVTANV																		
ORF1ab 5140	NSP12 RdRp	YLRKHFSMM									128 nM	134 nM	4 nM	47 nM						
ORF1ab 5141	NSP12 RdRp	LRKHFSMMI													313 nM					
ORF1ab 5142	NSP12 RdRp	RKHFSMMIL														310 nM				
ORF1ab 5143	NSP12 RdRp	KHFSMMILS																		
ORF1ab 5144	NSP12 RdRp	HFSMMILSD																		
ORF1ab 5145	NSP12 RdRp	FSMMILSDD																		
ORF1ab 5177	NSP12 RdRp	VLYYQNNVF												25 nM						
ORF1ab 5178	NSP12 RdRp	LYYQNNVFM																		
ORF1ab 5179	NSP12 RdRp	YYQNNVFMS																		
ORF1ab 5180	NSP12 RdRp	YQNNVFMSE																		
ORF1ab 5196	NSP12 RdRp	DLTKGPHEF																		
ORF1ab 5197	NSP12 RdRp	LTKGPHEFC																		
ORF1ab 5198	NSP12 RdRp	TKGPHEFCS																		
ORF1ab 5199	NSP12 RdRp	KGPHEFCSQ																		
ORF1ab 5200	NSP12 RdRp	GPHEFCSQH																		
ORF1ab 5201	NSP12 RdRp	PHEFCSQHT																		
ORF1ab 5202	NSP12 RdRp	HEFCSQHTM														54 nM	10 nM			
ORF1ab 5203	NSP12 RdRp	EFCSQHTML																		
ORF1ab 5204	NSP12 RdRp	FCSQHTMLV																		
ORF1ab 5205	NSP12 RdRp	CSQHTMLVK							227 nM											
ORF1ab 5217	NSP12 RdRp	DYVYLPYPD																		
ORF1ab 5218	NSP12 RdRp	YVYLPYPDP																		
ORF1ab 5219	NSP12 RdRp	VYLPYPDPS																		
ORF1ab 5220	NSP12 RdRp	YLPYPDPSR																		
ORF1ab 5221	NSP12 RdRp	LPYPDPSRI																		
ORF1ab 5222	NSP12 RdRp	PYPDPSRIL																		
ORF1ab 5223	NSP12 RdRp	YPDPSRILG																		
ORF1ab 5224	NSP12 RdRp	PDPSRILGA																		
ORF1ab 5225	NSP12 RdRp	DPSRILGAG																		
ORF1ab 5226	NSP12 RdRp	PSRILGAGC																		
ORF1ab 5227	NSP12 RdRp	SRILGAGCF																		
ORF1ab 5228	NSP12 RdRp	RILGAGCFV						153 nM												
ORF1ab 5229	NSP12 RdRp	ILGAGCFVD																		
ORF1ab 5230	NSP12 RdRp	LGAGCFVDD																		
ORF1ab 5248	NSP12 RdRp	IERFVSLAI															221 nM			
ORF1ab 5249	NSP12 RdRp	ERFVSLAID																		
ORF1ab 5250	NSP12 RdRp	RFVSLAIDA																		
ORF1ab 5251	NSP12 RdRp	FVSLAIDAY									477 nM			223 nM						
ORF1ab 5252	NSP12 RdRp	VSLAIDAYP																		
ORF1ab 5253	NSP12 RdRp	SLAIDAYPL						21 nM								487 nM				
ORF1ab 5349	NSP13 Hel	LCCKCCYDH																		
ORF1ab 5350	NSP13 Hel	CCKCCYDHV																		
ORF1ab 5371	NSP13 Hel	PYVCNAPGC																		
ORF1ab 5372	NSP13 Hel	YVCNAPGCD																		
ORF1ab 5373	NSP13 Hel	VCNAPGCDV																		
ORF1ab 5387	NSP13 Hel	LYLGGMSYY																		
ORF1ab 5388	NSP13 Hel	YLGGMSYYC						89 nM												
ORF1ab 5450	NSP13 Hel	CTERLKLFA					303 nM													
ORF1ab 5451	NSP13 Hel	TERLKLFAA																		
ORF1ab 5452	NSP13 Hel	ERLKLFAAE																		
ORF1ab 5453	NSP13 Hel	RLKLFAAET																		
ORF1ab 5559	NSP13 Hel	LSAPTLVPQ																		
ORF1ab 5560	NSP13 Hel	SAPTLVPQE																		
ORF1ab 5605	NSP13 Hel	QGPPGTGKS																		
ORF1ab 5606	NSP13 Hel	GPPGTGKSH																		
ORF1ab 5634	NSP13 Hel	SHAAVDALC																		
ORF1ab 5635	NSP13 Hel	HAAVDALCE																		
ORF1ab 5636	NSP13 Hel	AAVDALCEK																		
ORF1ab 5637	NSP13 Hel	AVDALCEKA																		
ORF1ab 5656	NSP13 Hel	RIIPARARV																		
ORF1ab 5657	NSP13 Hel	IIPARARVE																		
ORF1ab 5658	NSP13 Hel	IPARARVEC										213 nM								
ORF1ab 5716	NSP13 Hel	RAKHYVYIG																		
ORF1ab 5717	NSP13 Hel	AKHYVYIGD																		
ORF1ab 5718	NSP13 Hel	KHYVYIGDP																		
ORF1ab 5719	NSP13 Hel	HYVYIGDPA																		
ORF1ab 5720	NSP13 Hel	YVYIGDPAQ																	312 nM	
ORF1ab 5721	NSP13 Hel	VYIGDPAQL								206 nM										
ORF1ab 5722	NSP13 Hel	YIGDPAQLP																		
ORF1ab 5723	NSP13 Hel	IGDPAQLPA																		
ORF1ab 5724	NSP13 Hel	GDPAQLPAP																		
ORF1ab 5725	NSP13 Hel	DPAQLPAPR																		
ORF1ab 5771	NSP13 Hel	EIVDTVSAL									16 nM					298 nM			463 nM	
ORF1ab 5772	NSP13 Hel	IVDTVSALV						114 nM												
ORF1ab 5773	NSP13 Hel	VDTVSALVY																		
ORF1ab 5775	NSP13 Hel	TVSALVYDN																		
ORF1ab 5776	NSP13 Hel	VSALVYDNK																		
ORF1ab 5777	NSP13 Hel	SALVYDNKL																		
ORF1ab 5778	NSP13 Hel	ALVYDNKLK																		
ORF1ab 5779	NSP13 Hel	LVYDNKLKA																		
ORF1ab 5832	NSP13 Hel	KAVFISPYN																		
ORF1ab 5833	NSP13 Hel	AVFISPYNS																		
ORF1ab 5834	NSP13 Hel	VFISPYNSQ																		
ORF1ab 5835	NSP13 Hel	FISPYNSQN																		
ORF1ab 5855	NSP13 Hel	QTVDSSQGS																		
ORF1ab 5856	NSP13 Hel	TVDSSQGSE																		
ORF1ab 5857	NSP13 Hel	VDSSQGSEY																		
ORF1ab 5858	NSP13 Hel	DSSQGSEYD																		
ORF1ab 5859	NSP13 Hel	SSQGSEYDY																		
ORF1ab 5860	NSP13 Hel	SQGSEYDYV																		
ORF1ab 5861	NSP13 Hel	QGSEYDYVI																		
ORF1ab 5862	NSP13 Hel	GSEYDYVIF																		
ORF1ab 5880	NSP13 Hel	CNVNRFNVA																		
ORF1ab 5881	NSP13 Hel	NVNRFNVAI																		
ORF1ab 5882	NSP13 Hel	VNRFNVAIT																		
ORF1ab 5883	NSP13 Hel	NRFNVAITR													65 nM					
ORF1ab 5884	NSP13 Hel	RFNVAITRA																		
ORF1ab 5885	NSP13 Hel	FNVAITRAK																		
ORF1ab 6031	NSP14 ExoN	PLQLGFSTG																		
ORF1ab 6198	NSP14 ExoN	DAIMTRCLA																		
ORF1ab 6199	NSP14 ExoN	AIMTRCLAV						29 nM					24 nM							
ORF1ab 6307	NSP14 ExoN	CLFWNCNVD																		
ORF1ab 6320	NSP14 ExoN	NSIVCRFDT																		
ORF1ab 6321	NSP14 ExoN	SIVCRFDTR																		
ORF1ab 6322	NSP14 ExoN	IVCRFDTRV																		
ORF1ab 6323	NSP14 ExoN	VCRFDTRVL																		
ORF1ab 6341	NSP14 ExoN	GGSLYVNKH																		
ORF1ab 6342	NSP14 ExoN	GSLYVNKHA																		
ORF1ab 6343	NSP14 ExoN	SLYVNKHAF											279 nM	154 nM						
ORF1ab 6344	NSP14 ExoN	LYVNKHAFH																		
ORF1ab 6345	NSP14 ExoN	YVNKHAFHT																		
ORF1ab 6346	NSP14 ExoN	VNKHAFHTP																		
ORF1ab 6347	NSP14 ExoN	NKHAFHTPA																		
ORF1ab 6389	NSP14 ExoN	DYVPLKSAT																		
ORF1ab 6390	NSP14 ExoN	YVPLKSATC																		
ORF1ab 6391	NSP14 ExoN	VPLKSATCI																		
ORF1ab 6392	NSP14 ExoN	PLKSATCIT																		
ORF1ab 6393	NSP14 ExoN	LKSATCITR																		
ORF1ab 6394	NSP14 ExoN	KSATCITRC																331 nM		
ORF1ab 6395	NSP14 ExoN	SATCITRCN																		
ORF1ab 6396	NSP14 ExoN	ATCITRCNL																		
ORF1ab 6397	NSP14 ExoN	TCITRCNLG																		
ORF1ab 6398	NSP14 ExoN	CITRCNLGG																		
ORF1ab 6399	NSP14 ExoN	ITRCNLGGA																		
ORF1ab 6400	NSP14 ExoN	TRCNLGGAV																		
ORF1ab 6401	NSP14 ExoN	RCNLGGAVC																		
ORF1ab 6682	NSP15 NendoU	YAFEHIVYG																	177 nM	
ORF1ab 6698	NSP15 NendoU	GGLHLLIGL																		
ORF1ab 6746	NSP15 NendoU	VIDLLLDDF																		
ORF1ab 6747	NSP15 NendoU	IDLLLDDFV																		
ORF1ab 6839	NSP16 O-MT	MMNVAKYTQ																		
ORF1ab 6840	NSP16 O-MT	MNVAKYTQL											259 nM							
ORF1ab 6841	NSP16 O-MT	NVAKYTQLC																		
ORF1ab 6842	NSP16 O-MT	VAKYTQLCQ																		
ORF1ab 6843	NSP16 O-MT	AKYTQLCQY																		
ORF1ab 6844	NSP16 O-MT	KYTQLCQYL								139 nM										
ORF1ab 6845	NSP16 O-MT	YTQLCQYLN																		
ORF1ab 6846	NSP16 O-MT	TQLCQYLNT																		
ORF1ab 6869	NSP16 O-MT	GAGSDKGVA																		
ORF1ab 6870	NSP16 O-MT	AGSDKGVAP																		
ORF1ab 6871	NSP16 O-MT	GSDKGVAPG																		
ORF1ab 6872	NSP16 O-MT	SDKGVAPGT																		
ORF1ab 6922	NSP16 O-MT	WDLIISDMY																		
ORF1ab 6923	NSP16 O-MT	DLIISDMYD																		
ORF1ab 6924	NSP16 O-MT	LIISDMYDP																		
ORF1ab 6943	NSP16 O-MT	SKEGFFTYI																		
ORF1ab 6958	NSP16 O-MT	KLALGGSVA																		
ORF1ab 6959	NSP16 O-MT	LALGGSVAI																	11 nM	
ORF1ab 6960	NSP16 O-MT	ALGGSVAIK							110 nM											
ORF1ab 6961	NSP16 O-MT	LGGSVAIKI																		
ORF1ab 6962	NSP16 O-MT	GGSVAIKIT																		
ORF1ab 6963	NSP16 O-MT	GSVAIKITE																		
ORF1ab 6973	NSP16 O-MT	SWNADLYKL																		
ORF1ab 6974	NSP16 O-MT	WNADLYKLM																		
ORF1ab 6993	NSP16 O-MT	TNVNASSSE																		
ORF1ab 6998	NSP16 O-MT	SSSEAFLIG																		
ORF1ab 7024	NSP16 O-MT	HANYIFWRN																		
N 106	Nucleocapsid	PRWYFYYLG																		
N 107	Nucleocapsid	RWYFYYLGT																		
N 108	Nucleocapsid	WYFYYLGTG																		
N 109	Nucleocapsid	YFYYLGTGP																		

In Table 1 (for Excel format see *Extended data*) it is shown that there are >200 linear epitopes of 9 aa that are identical between SARS-CoV-2 and at least one of the common human coronaviruses, most of them with OC43 and HKU1 which, like SARS-CoV-2, belong to the group II coronaviruses. In a simplified model, if people would have been exposed to many of these epitopes through common HCoV infections, this kind of equals immunization by a small intracellular protein under natural viral infection conditions. Whereas live virus is commonly considered the gold standard in regard to inducing strong immunity, unless the virus has some tricks up its sleeve to manipulate the immune system, which for common human coronaviruses is not well investigated, a research grant proposal suggesting this as a vaccination strategy would probably fail. Reviewers of such proposal would righteously point out that the strategy would not induce neutralizing antibodies, which for combating some viral infections can be very important, and that for inducing MHC-I-restricted cell-mediated cytotoxicity memory, ideally, a much larger protein or more proteins should be taken. Those reviewers would conclude that for such small intracellular protein to induce strong immune memory it would be too dependent on the MHC alleles of the immunized person and would need too much luck in regard to immunogenicity. Nevertheless, those reviewers would probably also agree that in most persons thus vaccinated some (small) level of immune memory protection would be established, even with such small non-surface protein (e.g. Polakos *et al.*, 2001; Wasmoen *et al.*, 1995; Zhao *et al.,* 2005). Regardless of that this obviously is not the ideal way to induce a population-wide strong protective immunity (see the spread of COVID-19), together with other factors such as health and the number of encountered viruses (the strength of the viral challenge), the induced immune memory could make a difference for whether a person gets sick; at the population scale, it so may somewhat reduce the virus reproduction number. Importantly, by stimulating this HCoV-derived MHC-I restricted immune memory by vaccination (see below), it may become a more significant helper in fighting COVID-19.

### Software predictions of MHC-I-binding epitopes

Based on combinations of experimental results and computer learning, various software has been created that with some degree of reliability can predict how efficiently peptides can bind to the grooves of various MHC-I alleles. In the present study, we used the artificial neural network (ANN) function ( Lundegaard *et al.*, 2008) of the IEDB Analysis Resource ( http://tools.immuneepitope.org/mhci/) ( Dhanda *et al.*, 2019) which may achieve >75% reliability for predicting binding ( Lundegaard *et al.*, 2008). The software designers state that IC50 values of <50 nM and <500 nM are considered high and intermediate affinity, respectively, and are found for most epitopes known to stimulate cytotoxic T cells. Therefore, Table 1 only indicates the predicted IC50 values if lower than 500 nM. Table 1 shows these expected affinities for fourteen MHC-I alleles that are rather representative for sets of MHC-I alleles with similar binding properties (supertypes) and so represent a large part of the human MHC-I binding repertoire ( Doytchinova *et al.*, 2004; Lund *et al.*, 2004): HLA-A*0101 (supertype A1), HLA-A*0201 (A2), HLA-A*0301 (A3), HLA-A*2402 (A24), HLA-A*2601(A26), HLA-B*0702 (B7), HLA-B*0801 (B8), HLA-B*1501 (B62), HLA-B*2705 (B27), HLA-B*3901 (B39), HLA-B*4001 (B44), HLA-B*5801 (B58), HLA-C*0303 (C1), and HLA-C*0401 (C4). It is of note that Li *et al.* (2008) found that a SARS-CoV-1 15 aa peptide sequence (their “Replicase 4701-4715” peptide) encompassing the SARS-CoV-2/HCoV-shared ORF1ab4725 and ORF1ab4726 epitopes that are predicted to bind well to the MHC-I alleles HLA-A*0201 and HLA-B*3901 (see our Table 1) was associated with a CD8 ^+^ T cell response against SARS-CoV-1 in humans. However, Li *et al.* (2008) also found such CD8 ^+^ T cell response associated with a SARS-CoV-1 15 aa peptide (their “Nucleocapsid 106-120” peptide) encompassing the SARS-CoV-2/HCoV-shared N 106, N 107, N 108, and N 109 epitopes for which our analyses did not predict MHC-I binding (see our Table 1).

The MHC-I binding affinity is considered the most selective in determining which peptides are presented, but also steps in the peptide processing and loading pathways may play selective roles which are difficult to capture in prediction software ( Nielsen *et al.*, 2005). We argue that, if such steps would be selective for presentation, in most cases they would probably not differentiate between the 9 aa epitope in the SARS-CoV-2 context versus the respective HCoV context, since most of those epitopes are within stretches that also show many similarities in the neighboring residues ( *Extended data*).

Not all stable complexes of MHC-I with non-self peptides elicit a strong immune response, but “immunogenicity” features are hard to predict with meaningful reliability by *in silico* analysis ( Calis *et al.*, 2013), and in the present study we refrain from such predictions. Table 1 should, foremost, be understood as evidence of principle and a list of promising peptides, whereas only future experiments can prove MHC-I-mediated immune memory involving these or other peptides.

In regard to SARS-CoV-2 recognition, the common human coronaviruses may also induce some MHC-II-mediated immune memory by CD4 ^+^ helper T cells (as an example for shared epitope use by different coronaviruses see Zhao *et al.*, 2016). CD4 ^+^ helper T cells can help stimulate cells involved in antibody or cell-mediated cytotoxic immune responses ( Neefjes *et al.*, 2011). However, for this topic, in the present article, we have refrained from detailed (software) predictions because comparison of MHC-II epitopes across different viruses is harder than for MHC-I epitopes. Namely, although the core of MHC-II bound peptides is also only 9 aa, the surrounding amino acids are also part of the bound peptide that tends to be 12–25 aa ( Brown *et al.*, 1993; Rammensee *et al.*, 1995; Stern & Wiley, 1994) and can affect how the peptide interacts with the receptors on the CD4 ^+^ helper T cells ( Arnold *et al.*, 2002).

### Vaccination potential

Immune memory means that a secondary immune response, upon renewed encounter with the same pathogen, is faster and stronger than the primary immune response during the first encounter with the pathogen. This is based on expansion of specific B and T cell clones, which specifically recognize pathogen(-derived) epitopes, with some of those cells becoming memory cells ( Paul, 2013). This principle also causes that for a booster vaccination/immunization the requirements for efficiently inducing an immune response are lower than for a first vaccination/immunization (e.g. Du *et al.*, 2008; Goding, 1996; Schulze *et al.*, 2008). Especially in elderly people, who have a decreased ability to mount adaptive immune responses against new antigens, vaccination that stimulates an immune memory response may be beneficial ( Kaml *et al.*, 2006; Reber *et al.*, 2012; Wagner & Weinberger, 2020). As discussed above, people’s past infections with common coronaviruses probably did not induce a B cell memory for making antibodies that can neutralize SARS-CoV-2. However, as the current study shows by analysis of linear 9 aa epitopes, these common human coronaviruses are expected to induce CD8 ^+^ T cells that may potentially kill SARS-CoV-2-infected cells and so can help eradicate the virus. There are several possible ways to exploit this probable immune memory. For example, if using RNA for immunization ( Cohen, 2020), it may be best to also include SARS-CoV-2 genes that encode MHC-I epitopes that match those of the common coronaviruses. Alternatively, delivery of these epitopes to the MHC-I presentation system may be tried by peptide or protein based vaccines (e.g. Kohyama *et al.*, 2009; Slingluff, 2011; van Montfoort *et al.*, 2014; Yadav *et al.*, 2014), possibly in combination with some of the strategies that are currently being explored for non-specific stimulation of the immune system against COVID-19 ( Kupferschmidt & Cohen, 2020). Protein (-coding) vaccines, for example encompassing a large part of the SARS-CoV-2 ORF1ab product, would have an advantage over peptide-vaccines by including multiple possible MHC-I and also MHC-II epitopes, and be less dependent on MHC-allele matching and the quality of software predictions. Naturally, as for any new vaccine strategy, it should be carefully assessed whether the benefits of the induced type of immunity outweigh the potential deleterious health effects caused by, for example, an increased inflammation response ( Cohen, 2020; Weingartl *et al.*, 2004). Another fundamental concern is the maximum level of protection that can be generated by vaccination against coronavirus infections in humans, considering that infection of volunteers with HCoV-229E live virus gave only partial protection upon infection with the same virus one year later ( Callow *et al.*, 1990). Additional questions specifically related to the contents of our study are whether the history of previous—especially recent—infections with common coronaviruses, or people’s MHC alleles, affect people’s resistance to SARS-CoV-2. Most definitely, if discussing possible strategies for vaccination against SARS-CoV-2, pre-existing MHC-I-based immunity derived from previous infections with common coronaviruses should be part of the consideration.

## Notifications

Although we were not aware of this at the time of writing, a recent paper appeared with overlapping contents ( Nguyen *et al.*, 2020). The Nguyen *et al.* study was more complete on SARS-CoV-2 MHC epitope predictions and made an association with global MHC allele distributions. The advantage of our study is a more concentrated focus on the MHC-I mediated memory expected from previous coronavirus infections, and the vaccination potential deriving from that memory.

After we had submitted our study, two studies reported *in vitro* responses of T cells against SARS-CoV-2 peptides, which might represent memory from previous infections with common coronaviruses ( Braun *et al.*, 2020; Grifoni *et al.*, 2020). However, both studies only used peptide mixes without identifying the responsible peptide, and at least several of the observed responses necessitated the allowance of peptide ligand sequence mismatches for T cell receptor to MHC/peptide binding (T cell cross-reactivity). Negative control donors, who with certainty had never been infected with common coronaviruses, were not available for the experiments, and conclusions that the observed responses were from T cell memory from previous coronavirus infections, and have *in vivo* relevance, should be considered only cautiously. Discussion of this topic is important because the two studies concluded a potential of the common coronavirus S proteins to induce CD4 ^+^ T cell memory ( Braun *et al.*, 2020; Grifoni *et al.*, 2020) and CD8 ^+^ T cell memory ( Grifoni *et al.*, 2020), whereas these proteins do not share 9 aa identical stretches with SARS-CoV-2 (see our article and Supplementary Fig. 1 in Braun *et al.*, 2020), and would arguably necessitate the allowance of peptide sequence mismatches (T cell cross-reactivity) for inducing an efficient MHC-mediated T cell response. As we pointed out in our article, although SARS-CoV-2 S protein is the prime vaccine component candidate for inducing neutralizing antibodies, for a more realistic chance to efficiently boost existing T cell memory it probably would be better to additionally include other SARS-CoV-2 proteins that do share identical MHC epitopes with common coronaviruses.

Regarding the potential of existing CD8 ^+^ T cell memory cells to help fight COVID-19 disease, a recent observation by Liao *et al.,* (2020) might be interesting. Their study suggests that in COVID-19 patients with pneumonia, ZNF683 ^+^ CD8 ^+^ T cell clonal expansion may protect the patient from more severe disease.

## Data availability

### Underlying data

Severe acute respiratory syndrome coronavirus 2 isolate Wuhan-Hu-1, complete genome, Accession number MN908947: https://www.ncbi.nlm.nih.gov/nuccore/MN908947

Human coronavirus OC43, complete genome, Accession number NC_005147.1: https://www.ncbi.nlm.nih.gov/nuccore/NC_005147.1?report=genbank

Human coronavirus HKU1, complete genome, Accession number NC_006577: https://www.ncbi.nlm.nih.gov/nuccore/NC_006577

Human coronavirus 229E, complete genome, Accession number NC_002645: https://www.ncbi.nlm.nih.gov/nuccore/NC_002645

Human Coronavirus NL63, complete genome, Accession number NC_005831: https://www.ncbi.nlm.nih.gov/nuccore/NC_005831

### Extended data

Harvard Dataverse: Extended data. Sequence alignments of SARS-CoV-2 ORF1ab and N proteins with their counterparts in the common human coronaviruses, https://doi.org/10.7910/DVN/CNPUPA ( Dijkstra, 2020a).

Harvard Dataverse: Excel format version of Table 1. https://doi.org/10.7910/DVN/LOBKLV ( Dijkstra, 2020b).

Data are available under the terms of the Creative Commons Zero "No rights reserved" data waiver (CC0 1.0 Public domain dedication).
