# Supplementary material for: Expected immune recognition of COVID-19 virus by memory from earlier infections with common coronaviruses in a large part of the world population
Source: F1000Res. 2020 Jul 17;9:285. Originally published 2020 Apr 23. [Version 2] doi: 10.12688/f1000research.23458.2 (PMC7309412; doi:10.12688/f1000research.23458.2)
Supplement: Peer review history

# Peer review: Reviewer response for version 1

**DOI:** 10.5256/f1000research.25889.r63246

**Published:** 2020-06-22

**Authors:** Andrea Sant

**Affiliations:** 1 Department of Microbiology and Immunology, University of Rochester Medical Center, Rochester, NY, 14642, USA; 2 David H. Smith Center for Vaccine Biology and Immunology, Rochester, NY, USA

These analyses of potential cross reactive CD8 T cell epitopes between the current SARS-CoV-2 and “seasonal” endemic human CoV is useful and timely and the discussion is balanced.

 There are several modifications that I believe would improve the clarify and value of the manuscript.

 Based on the first sentence of the paragraph entitled “the possibility of matching linear epitopes…”, the authors sate that the two major arms of immune memory…are antibody and CD8 T cells” I believe this is incorrect, as CD4 T cells can directly impact lung pathology and contribute to both protective and pathological immune responses. In fact a recent paper uploaded to BioRxiv suggested that it was populations in the CD4 T cell compartments that correlated with disease severity. The authors should acknowledge that all three subsets of the adaptive response (B cells, CD8 and CD4 T cells) are likely to be important, but this manuscript focusses on CD8 epitopes.

 The authors refer to the “software owners” when describing cutoffs. They are perhaps better described as software “designers”.

 When discussing “Vaccine Potential”, the authors state that the secondary response is “faster and stronger”. This should be more accurately described, with some references, in a way that points out the higher frequency of responding cells during memory recall, and lower thresholds of TcR engagement needed for T cell activation, both qualities that contribute to a competitive advantage of memory cells.

 Because the nature of CD8 memory to the different antigens screened by the authors is not known, the epitopes identified may or may not be targets of cross reactive memory recall. Therefore, the word “expected” should be substituted for “Potential” or some other word that indicates that the epitope list includes candidates but not expected epitopes.

 I think the Table could be made quite a lot smaller and thus more valuable to the reader. The source proteins could be indicated as an abbreviation provided in the legend as could the various seasonal strains. The boxes could then be quite small, and either be positive or negative. In any case, an effort should be made to condense this table.

Is the work clearly and accurately presented and does it cite the current literature?

Yes

If applicable, is the statistical analysis and its interpretation appropriate?

Yes

Are all the source data underlying the results available to ensure full reproducibility?

Yes

Is the study design appropriate and is the work technically sound?

Yes

Are the conclusions drawn adequately supported by the results?

Partly

Are sufficient details of methods and analysis provided to allow replication by others?

Yes

Reviewer Expertise:

Immunology

I confirm that I have read this submission and believe that I have an appropriate level of expertise to confirm that it is of an acceptable scientific standard.

# Response

**Published:** 2020-07-08

**Authors:** Johannes M. Dijkstra

**Affiliations:** Fujita Health University, Japan

Dear Dr. Sant,

 Thank you for reviewing our article. We highly appreciate your comment that our article is timely and well balanced. Especially the latter is important, since we did not want to make a general audience too enthusiastic, while we simultaneously wanted to stress that there is a chance that this MHC-I-mediated immunity might (possibly after boostering by vaccines) give real protection.

 You are correct that immune memory by CD4 ^+^ cells is not only relevant to their control of B cell and CD8 T cell responses, and we have rewritten the sentence and paragraph on the “major arms of immune memory.” As you state, our paper focusses on CD8 ^+^ T cell memory indeed, because that is the memory response that can be predicted most reliably by sequence analysis alone. In the notification section we now shortly discuss two papers that appeared after we submitted our paper, and which claim to have found SARS-CoV-2 recognizing CD4 ^+^ and CD8 ^+^ T cell memory derived from previous common coronavirus infections.

 As requested, we have now changed “software owners” to “software designers.”

 As requested, we now have added more references of an enhanced immune reaction after a second (booster) immunization. However, we do not feel that for our type of paper it is necessary to discuss the mechanisms of memory T cells besides just mentioning their involvement.

 As for the words “expected” versus “potential.” We feel that we used the word “expected” correctly. Table 1, where all the peptides are listed, carries neither of these two words and has a very neutral title. In the text, some peptides are referred to as “expected” to bind a particular MHC molecule, a term clearly relating to the indicated software and literature. Given the large number of potential MHC-I epitopes shared between the viruses, we “expect” previous common coronavirus infections to have induced some CD8+ T cell immune memory that recognizes SARS-CoV-2; this claim is not about the protective value of this memory, and we feel, therefore, that the word “expect” is within reasonability.

 As for the Table format. The format was chosen by the journal editorial team, and we can see that for some uses it has advantages. However, we understand your concern, and now have added an Excel format variant of the Table to the supplement section so that readers can more easily view and interact with the data.

 Apart from addressing the reviewer’s comments, we corrected a mistake and now informed the readers that there is a single 8 aa match between compared S proteins.

 Apart from addressing the reviewer’s comments, we now also added the information that the study by Callow *et al.* (1990) on HCoV-229E, concluded imperfect immune memory protection even by live virus infection one year before challenge.

 We are aware of the time and effort reviewing takes, and are very thankful of your thoughtful contribution. It lifts our spirits that you and the other reviewers consider our article a nice contribution to the COVID-19 studies.

 Sincerely,

 also on behalf of Keiichiro Hashimoto

 Hans (J.M.) Dijkstra

# Peer review: Reviewer response for version 1

**DOI:** 10.5256/f1000research.25889.r63655

**Published:** 2020-06-22

**Authors:** Liisa K. Selin, Anna Gil

**Affiliations:** 1 University of Massachusetts Medical School, Worcester, MA, USA; 2 Department of Pathology, University of Massachusetts Medical School, Worcester, MA, USA

This manuscript reports a very useful study that extends our knowledge of peptide-MHC recognition by CD8+ T cells during emerging virus infections such as SARS-CoV-2. Detailed *in silico* analysis showed the presence of potential epitopes shared between new types of  betacoronavirus: SARS-CoV-2 and common human alphacoronaviruses: OC43, HKU1, 229E and NL63. Due to the high prevalence of the common coronaviruses authors suggest that the large part of the human population has already some degree of specific memory T cell response before having been infected with the virus.

 As authors already mentioned in their manuscript the similar study by Nguyen A. et al (JVI, 2020 ^1^) demonstrated the HLA binding affinity of all possible 8- to 12-mers from SARS-CoV-2 proteome. This group found that HLA-B15:03 type has the greatest capacity to present highly conserved peptides which are shared among coronaviruses suggesting a cross-protective T cell immune response. In current manuscript using different prediction software authors identified and showed the sequence of epitopes which bind well to similar HLA type, HLA-B15:01. Interestingly, one of the epitopes (YLRKHFSM) can be bound by 4 different HLA types. The obvious strength of this study is the demonstration that certain epitopes, which are identical between SARS-CoV-2 and the common human coronaviruses are being predicted as high affinity binders in multiple HLA-A and B types.

 Overall, the work reports important new details about SARS-CoV-2  epitopes theoretically being recognized by human CD8+ T cells. Undeniably, future experiments can prove if generated memory immune responses are specific to the proposed epitopes.

 There are some suggestions:

1. The analysis of p/MHCI binding for HLA-C type (if available) would certainly complete the list of presented epitopes
2. The introduction part subtitled: ”The possibility of matching linear epitopes…” has missing information about previously published reports regarding T cell response in individuals infected with coronaviruses, either common or SARS-CoV.
3. In the discussion part readers may wonder why the authors did not discuss their  findings with those already published (although they may not have been out at the time of submission) but should be included in the revision.

Is the work clearly and accurately presented and does it cite the current literature?

Partly

If applicable, is the statistical analysis and its interpretation appropriate?

Yes

Are all the source data underlying the results available to ensure full reproducibility?

Yes

Is the study design appropriate and is the work technically sound?

Yes

Are the conclusions drawn adequately supported by the results?

Yes

Are sufficient details of methods and analysis provided to allow replication by others?

Yes

Reviewer Expertise:

T cel viral immunology

We confirm that we have read this submission and believe that we have an appropriate level of expertise to confirm that it is of an acceptable scientific standard.

## References

[1] : Human Leukocyte Antigen Susceptibility Map for Severe Acute Respiratory Syndrome Coronavirus 2. *J Virol*.2020;94(13) : 10.1128/JVI.00510-20 10.1128/JVI.00510-20 32303592PMC7307149

# Response

**Published:** 2020-07-08

**Authors:** Johannes M. Dijkstra

**Affiliations:** Fujita Health University, Japan

Dear Dr. Gil and Dr. Selin,

 Thank you for your kindness to review our article. We are glad that you find our study very useful, and that you agree with the conclusions. Coming from experts like you, this is very reassuring.

 We have now added a bit more information on the Nguyen *et al*. study in the Notification section. The advantage of the Nguyen *et al.* study was that they were more complete on SARS-CoV-2 MHC epitope predictions and made an association with MHC allele distributions. The advantage of our paper is a more concentrated focus on the memory expected from previous coronavirus infections, and the vaccination potential deriving from that memory. They were first, which we acknowledged, although we wrote our paper independent of their article, and during the submission process of our article theirs was not an indexed publication yet. Presumably, this type of overlap will happen a lot with a topic as intensively studied as coronavirus, and we feel we took a reasonable approach for dealing with their study. From your reviews, we understand that you and the other reviewer, Dr. Sant, find this within acceptability.

 Thank you for referring to the interesting YLRKHFSMMIL stretch which is identical between HCoV-NL63 and SARS-CoV-2, as indeed it harbors predicted binding epitopes for several MHC-I supertypes. However, we prefer not to discuss this in text form, because there are many uncertainties (e.g., about recent HCoV-NL63 distributions in the world population) and a textual discussion may not add clarity to the table presentation.

 Based on your advice, we now have added HLA-C predictions to Table 1.

 Likewise, we now have added a more extensive summary of previous reports on T cell memory after coronavirus infections.

 Apart from addressing the reviewer’s comments, we corrected a mistake and now informed the readers that there is a single 8 aa match between compared S proteins.

 Apart from addressing the reviewer’s comments, we now also added the information that the study by Callow *et al.* (1990) on HCoV-229E, concluded imperfect immune memory protection even by live virus infection one year before challenge.

 Again, we like to thank you for the reviewing, as we are aware of the time and effort that it takes. We are very happy that our article is appreciated, since it deals with such an important topic.

 Sincerely,

 also on behalf of Keiichiro Hashimoto

 Hans (J.M.) Dijkstra
